# Trends in evolution of the Triatomini tribe (Hemiptera, Triatominae): reproductive incompatibility between four species of *geniculatus* clade

**DOI:** 10.1186/s13071-022-05540-z

**Published:** 2022-11-02

**Authors:** Yago Visinho dos Reis, Jader de Oliveira, Fernanda Fernandez Madeira, Amanda Ravazi, Ana Beatriz Bortolozo de Oliveira, Denis Vinicius de Mello, Fabricio Ferreira Campos, Maria Tercília Vilela de Azeredo-Oliveira, João Aristeu da Rosa, Cleber Galvão, Kaio Cesar Chaboli Alevi

**Affiliations:** 1 Instituto de Biociências Rua Dr. Antônio Celso Wagner Zanin, Universidade Estadual Paulista “Júlio de Mesquita Filho” (UNESP), 250, Distrito de Rubião Júnior, Botucatu, SP 18618-689 Brasil; 2 Laboratório de Entomologia em Saúde Pública, Departamento de Epidemiologia, Faculdade de Saúde Pública, Universidade de São Paulo (USP), Av. Dr. Arnaldo 715, São Paulo, SP Brasil; 3 Laboratório de Biologia Celular, Instituto de Biociências, Universidade Estadual Paulista “Júlio de Mesquita Filho” (UNESP), Letras e Ciências Exatas, Rua Cristóvão Colombo 2265, São José Do Rio Preto, SP 15054-000 Brasil; 4 Laboratório de Parasitologia, Faculdade de Ciências Farmacêuticas, Universidade Estadual Paulista “Júlio de Mesquita Filho” (UNESP), Rodovia Araraquara-Jaú Km 1, 14801-902 Araraquara, SP Brasil; 5 Instituto Oswaldo Cruz (FIOCRUZ), Laboratório Nacional e Internacional de Referência em Taxonomia de Triatomíneos, Av. Brasil 4365, Pavilhão Rocha Lima, Sala 505, 21040-360 Rio de Janeiro, RJ Brasil

**Keywords:** Triatomines, Chagas disease vectors, *Panstrongylus*, *Nesotriatoma*, Prezygotic isolation barrier

## Abstract

**Background:**

The *geniculatus* clade, composed by the *rufotuberculatus*, *lignarius*, *geniculatus* and *megistus* groups, relates evolutionarily the species of the genus *Panstrongylus* and *Nesotriatoma*. Several studies have shown that triatomine hybrids can play an important role in the transmission of Chagas disease. Natural hybrids between species of the *geniculatus* clade have never been reported to our knowledge. Thus, carrying out experimental crosses between species of the *geniculatus* clade can help to elucidate the taxonomic issues as well as contribute to the epidemiological knowledge of this group.

**Methods:**

Experimental crosses were carried out between species of the *megistus* and *lignarius* groups to evaluate the reproductive compatibility between them. A phylogenetic reconstruction was also performed with data available in GenBank for the species of the *geniculatus* clade to show the relationships among the crossed species.

**Results:**

Phylogenetic analysis grouped the species of the *geniculatus* clade into four groups, as previously reported. In the interspecific crosses performed there was no hatching of eggs, demonstrating the presence of prezygotic barriers between the crossed species and confirming their specific status.

**Conclusions:**

In contrast to the other groups of the Triatomini tribe, as well as the Rhodniini, there are prezygotic barriers that prevent the formation of hybrids between species of the *megistus* and *lignarius* groups. Thus, the *geniculatus* clade may represent an important evolutionary model for Triatominae, highlighting the need for further studies with greater sample efforts for this clade (grouping the 17 species of *Panstrongylus* and the three of *Nesotriatoma*).

**Graphical Abstract:**

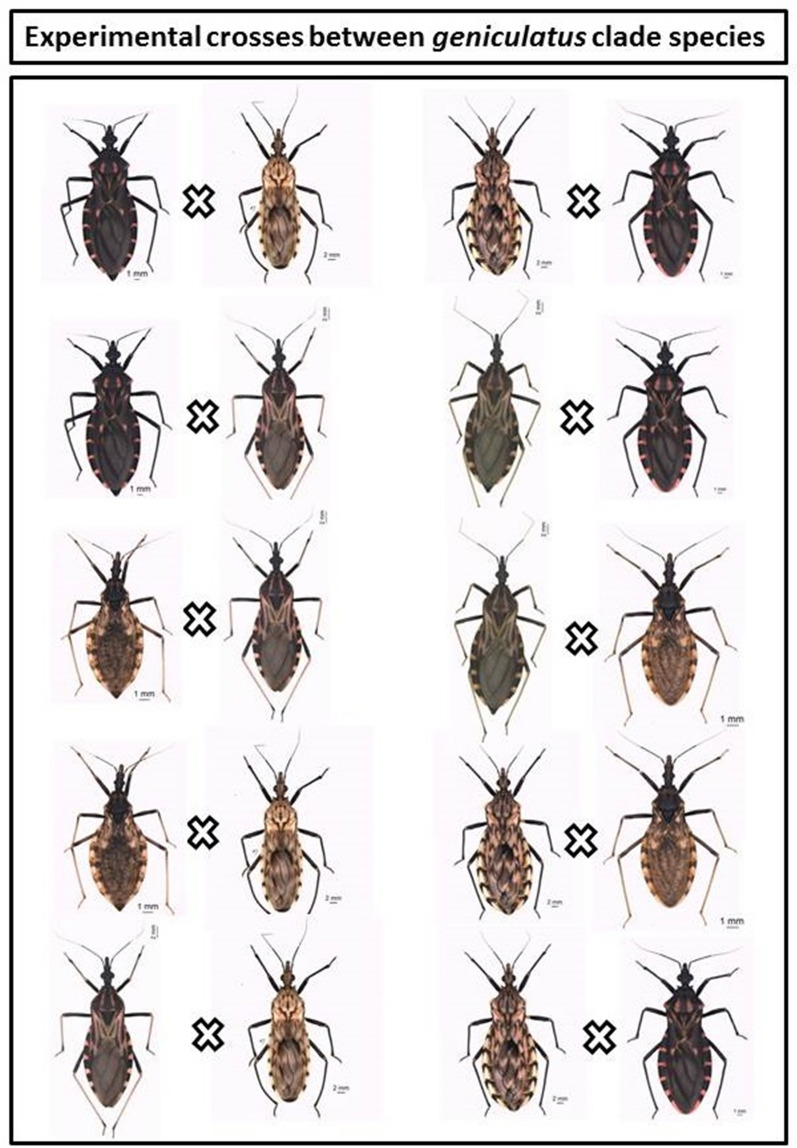

## Background

The triatomines (Hemiptera, Triatominae) are insects of great importance to public health because all 154-living species of the subfamily Triatominae [1–3] are considered potential vectors of the protozoan *Trypanosoma cruzi* (Chagas, 1909) (Kinetoplastida, Trypanosomatidae) etiological agent of Chagas disease [4]. This disease is neglected, has no cure in the chronic phase (effective treatment being only possible in the early stages of infection) and affects about 6 to 7 million people worldwide [5, 6]. In addition, about 120 million people live in endemic areas with risk of infection [6].

Currently, the subfamily Triatominae is divided into 18 genera and five tribes (Alberproseniini, Bolboderini, Cavernicolini, Rhodniini and Triatomini) [1, 7]. The Triatomini tribe is the most numerous (composed of 114 species grouped in ten genera [1–3]) and one of the most important from an epidemiological point of view [8]. Two most diverse genera in Triatomini (*Triatoma* Laporte, 1832, and *Panstrongylus* Berg, 1879) are paraphyletic [8, 9]; therefore, this tribe is divided into clades, groups, complexes and subcomplexes. Although these species groupings are not formally recognized as taxonomic ranks, Justi et al. [9] propose that they represent monophyletic lineages.

The *geniculatus* clade, composed by the *rufotuberculatus*, *lignarius*, *geniculatus* and *megistus* groups [10, 11], relates evolutionarily the species of the genus *Panstrongylus* and *Nesotriatoma* Usinger, 1944 [8–11]. The taxonomy of *Nesotriatoma* spp. is quite discussed because some authors consider *Nesotriatoma* a valid genus [1, 7, 9, 12–15], and others classify the species of this genus in *Triatoma* [8, 16–18]. However, phylogenetic studies indicate the validity of the genus *Nesotriatoma* and demonstrate that this genus is closer to *Panstrongylus* spp. [9]. Chromosomal data also support this relation [16, 19–21]. In addition, a new species [*N. confusa* Oliveira et al. (2018)] has recently been described from specimens that were incorrectly classified as *N. bruneri* Usinger, 1944 [15].

Natural hybrids between species of the *geniculatus* clade have never been reported. Recently Villacís et al. [22] performed experimental crosses between two species of the *rufotuberculatus* group [*P. chinai* (Del Ponte, 1929) and *P. howardi* (Neiva, 1911)] and observed the production of hybrids. Several studies have shown that triatomine hybrids can play an important role in the transmission of Chagas disease [23–26]. Shorter defecation time [23] and greater fitness [24, 25] have been observed in the hybrids resulting from crosses between *Triatoma* species of the *phyllosoma* complex compared to the parents. Higher fitness has also been reported for hybrids between *T. protracta* (Uhler, 1894) subspecies [26]. Thus, we consider that carrying out experimental crosses between species of the clade *geniculatus* can help to elucidate the taxonomic problems as well as contribute to the epidemiological knowledge of this group.

## Methods

### Phylogenetic analysis

Sequences of several molecular markers for 13 taxa available in GenBank (Table 1) were aligned in the MEGA 11 program [27] using the Muscle method [28]. The alignments were concatenated by name using the Seaview4 program [29], resulting in an alignment with 8617 nucleotides. The phylogenetic reconstruction was performed using Beast 1.8.4 [30] under the GTR + I + G model, a strick clock model and Yule Process prior [31, 32]. The analysis was carried out with a total of 100 million generations. Trees were sampled every 1000 generations and burn-in adjusted to 25%. Tracer v. 1.7 [33] was used to verify the stabilization (ESS values > 200) of the sampled trees. The generated phylogenetic tree was visualized and edited in the FigTree v.1.4.4 program [34] and Adobe Illustrator CS6.

**Table 1:** GenBank accession number for each marker used in the phylogenetic analysis

Species	Molecular markers								
	*16S*	*18S*	*28S*	*cytb*	*COI*	*COII*	ITS-1	ITS-2	*12S*
*Geniculatus* clade									
* P. chinai*	–	–	–	JX400960	–	–	–	AJ306547	–
* P. geniculatus*	AF394593	–	KX109907	KX109903	–	–	AM949585	AJ306543	–
* P. howardi*	–	–	–	JX400969	–	–	–	JX400871	–
* P. lignarius*	AY185833	JQ897584	KX109906	ON262111	AF449141	–	–	AJ306549	AY185818
* P. lutzi*	KC248969	–	KC249135	KC249227	KC249307	KC249401	ON262110	–	–
* P. megistus*	KC248975	AJ243336	KC249141	KC249232	KC249312	KC249403	AM949580	AJ306542	AF021178
* P. rufotuberculatus*	KY748239	AJ421955	–	JX400989	–	–	–	AJ306546	–
* P. tibiamaculatus*	KC249080	KC249127	KC249214	KC249296	KC249389	KC249485	ON262109	–	AY185829
* P. tupynambai*	KC248978	–	KC249142	KC249234	–	KC249404	–	–	–
* N. confusa*	KC248989	–	KC249146	–	–	KC249418	–	–	–
* N. flavida*	AY035451	AJ421959	–	JX848648	–	–	–	AM286732	–
Outgroup									
* T. brasiliensis*	KC248985	AJ421957	KC249145	KC249239	KC249318	KC249413	KJ125138	KJ125138	AF021187
* R. prolixus*	AF324519	AJ421962	AF435860	AF045718	AF449138	–	–	AJ286888	AF394519

### Experimental crosses

**Fig. 1:**
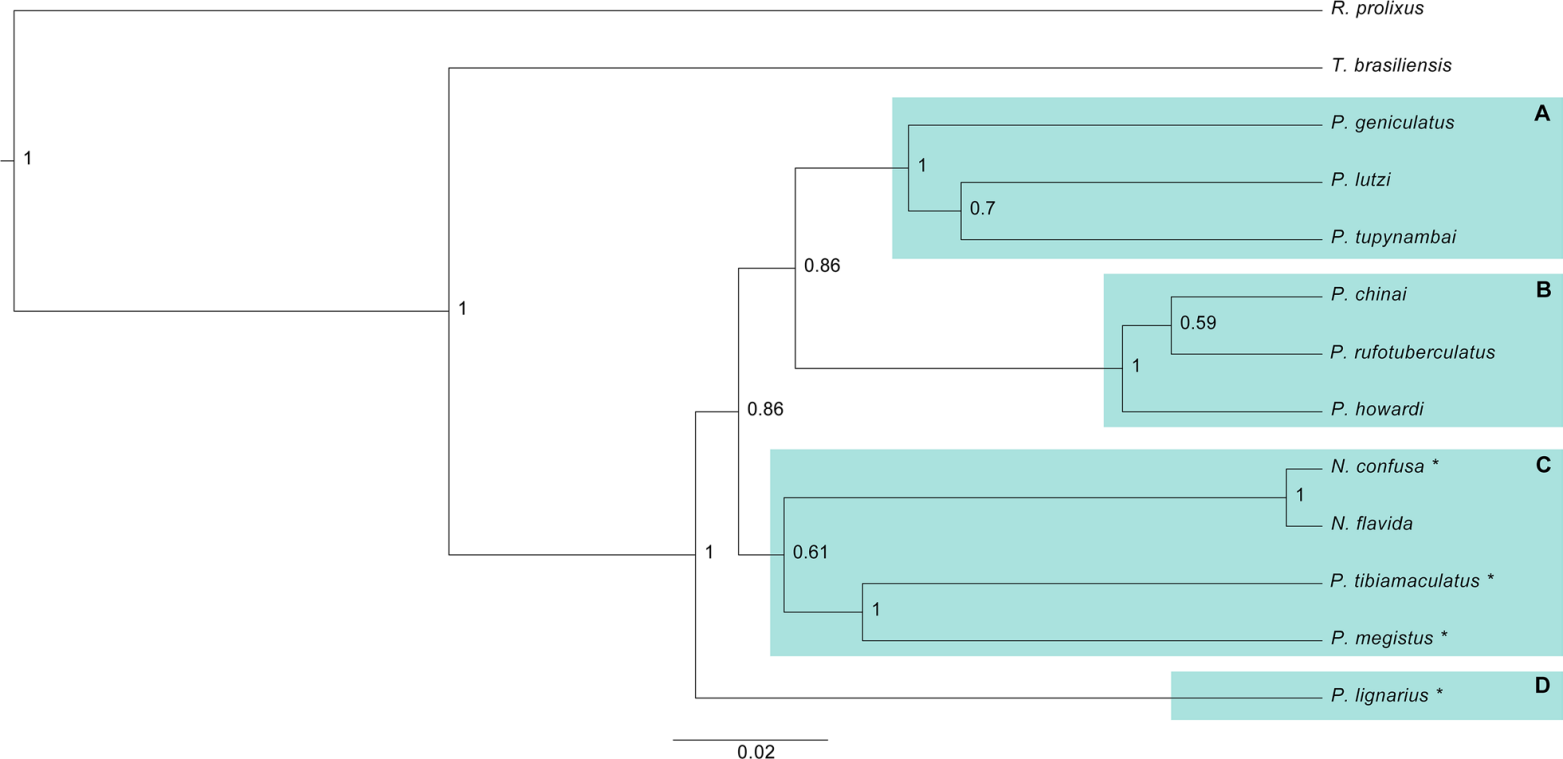
Bayesian phylogeny of *geniculatus* clade species. The posterior probability is shown in the nodes. **A** *Geniculatus* group. **B** *Rufotuberculatus* group. **C** *Megistus* group. **D** *Lignarius* group. *Species used in the experimental crosses

To evaluate the reproductive compatibility [35] between the species of the *geniculatus* clade, reciprocal crossing experiments were conducted among species of the genus *Panstrongylus* and *Nesotriatoma* (Table 2). Species were selected according to phylogenetic proximity (Fig. 1) and the availability of colonies at Triatominae insectary of the School of Pharmaceutical Sciences, São Paulo State University (FCFAR/UNESP), Araraquara, São Paulo, Brazil, where the experiments were carried out. The insects were sexed as fifth instar nymphs based on Rosa et al. [36]: the nymphs were separated from the colony and analyzed one by one under a stereoscopic microscope, with emphasis on the ninth segment of the sternite and tergite (characters that allow the differentiation between males and females). Posteriorly, males and females were kept separately until they reached the adult stage to cross adult virgins [37]. For the crosses, three couples from each set were placed in separate plastic jars (5 cm diameter × 10 cm height) and kept at room temperature (average of 24 ºC [38]) and an average relative humidity of 63% [38]. The crosses were maintained for 4 months. Weekly, the insects were fed on duck blood and the eggs were collected. Matings between species were observed only occasionally during the period of feeding and maintenance of crosses. The eggs were checked for 2 months after the end of the crosses to assess the hatching rate.

**Table 2:** Experimental crosses performed between *geniculatus* clade species

Crossing experiments	Number of eggs (mean ± SD)		Egg hatching rate (%)
Interspecific			
* P. megistus ♀* × *P. lignarius ♂*	157 (52 ± 28)		0
* P. lignarius ♀* × *P. megistus ♂*	523 (174 ± 152)		0
* P. megistus ♀* × *P. tibiamaculatus ♂*	107 (36 ± 4)		0
* P. tibiamaculatus ♀* × *P. megistus ♂*	265 (88 ± 2)		0
* P. lignarius ♀* × *P. tibiamaculatus ♂*	546 (182 ± 131)		0
* P. tibiamaculatus ♀* × *P. lignarius ♂*	68 (23 ± 9)		0
* N. confusa ♀* × *P. tibiamaculatus ♂*	111 (37 ± 6)		0
* P. tibiamaculatus ♀* × *N. confusa ♂*	164 (55 ± 9)		0
* P. lignarius ♀* × *N. confusa ♂*	122 (41 ± 3)		0
* N. confusa ♀* × *P. lignarius ♂*	130 (43 ± 7)		0
Intraspecific (control)			
* P. megistus*	372 (124 ± 87)		68
* P. lignarius*	700 (233 ± 12)		51
* P. tibiamaculatus* ^a^	190 (63 ± 4) ^a^		65 ^a^

*SD *standard deviation

^a^Neves et al.[39]

Furthermore, intraspecific crosses (Table 2) were also performed for control following the same methodology as for interspecific crosses. Unfortunately, intraspecific crosses of *N. confusa*, as well as interspecific crosses between *N. confusa* and *P. megistus* (Burmeister, 1835), were not performed because of the low population in the FCFAR/UNESP colony. The data used as control for *P. tibiamaculatus* (Pinto, 1926) were obtained from Neves et al. [39] (although Neves et al. [39] consider *P. tibiamaculatus* to be *T. tibiamaculata*, we highlight that this species was recently transferred to the genus *Panstrongylus* based on integrative taxonomy [11]).

## Results and discussion

In none of the interspecific crosses did the eggs hatch; in contrast, the hatching rate ranged from 51–68% in the intraspecific crosses (Table 2). Although some clades showed support < 0.8 (which highlights the importance of including more taxa and mainly new genes to rescue the natural history of the *geniculatus* clade), most clades were recovered with good support (later probability > 0.8). The *rufotuberculatus* and *geniculatus* groups were recovered as monophyletic (Fig. 1A and B). *Panstrongylus megistus* and *P. tibiamaculatus* were recovered as sister species, grouping with *Nesotriatoma* spp. (Fig. 1C). Already *P. lignarius* (Walker, 1873) is the most divergent species within the *geniculatus* clade (Fig. 1D). Thus, the species selected for the experimental crosses are close phylogenetically (with the exception of *P. lignarius*).

The phylogenetic relationships obtained in our analysis are very similar to the most recent phylogenies of this group [10, 11]. The previously proposed groups (*rufotuberculatus*, *lignarius*, *geniculatus* and *megistus* [10, 11]) were also recovered as monophyletic (Fig. 1). Thus, the presence of a prezygotic barrier observed between the crosses of *P. tibiamaculatus* with *P. lignarius* (Table 2) (both with 2n = 23 chromosomes [40]) may be associated with the divergence between these taxa, since they belong to distinct groups (Fig. 1). Until now, only Villacís et al. [22] had carried out experimental crosses in the genus *Panstrongylus*. The authors crossed two sister species of the *rufotuberculatus* group (*P. chinai* and *P. howardi*) that present morphological similarities and the same number of chromosomes (2n = 23) and observed the hatching of hybrids in the first generation (F1) (absence of prezygotic barrier). The hybrids reached the adult stage but were sterile (postzygotic barrier of sterility of the hybrid), confirming the specific status of the taxa, based on the biological species concept.

Absence of hybrids between *P. megistus* and other species of *geniculatus* clade is expected, mainly because this species presents a karyotype (2n = 21) [40] different from the other species of *Panstrongylus* (2n = 22, 23 and 24) [40, 41] and *Nesotriatoma* spp. (2n = 23) [40], and the number of chromosomes can act as a barrier of reproductive isolation for Triatomini tribe [39]. However, the absence of hybrids among the other crosses (Table 2) is an interesting and intriguing result for Triatomini tribe evolutionary studies, since experimental hybrids have already been observed for species that did not derive from an ancestor—for example, *T. infestans* (Klug, 1834) × *T. rubrovaria* (Blanchard, 1843), *T. maculata* (Erichson, 1848) × *T. sordida* (Stål, 1859), *T. maculata* × *T. infestans*, *T. maculata* × *T. brasiliensis* Neiva, 1911, and *T. pseudomaculata* Corrêa & Espínola, 1964 × *T. infestans* [42].

**Fig. 2:**
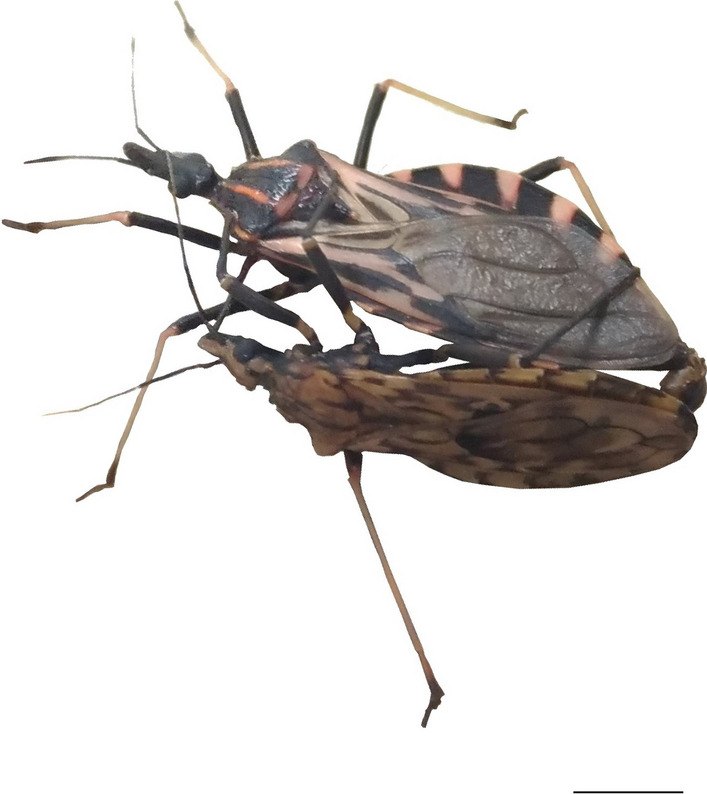
Interspecific mating observed between *Panstrongylus* spp. (*P. tibiamaculatus* ♀ × *P. lignarius* ♂). The background was removed with Adobe Photoshop CS6. Bar: 6 mm

The position of *Nesotriatoma* spp. in the clade *geniculatus* leads us to question whether *Nesotriatoma* would also be a *Panstrongylus* with homoplasy (as observed for *P. tibiamaculatus* [11]) because there is cytogenetic and phylogenetics evidence that confirms this relationship [9, 13, 16, 19]. The reproductive isolation observed between *N. confusa* and *geniculatus* clade species (Table 2) may be due to the long time these species have been geographically isolated, since *Nesotriatoma* spp. are found only in the Antillean Islands [8, 43]. It has been suggested that the ancestor of *Nesotriatoma* spp. reached these islands approximately 14.8–18.8 million years ago [8]. As the selective pressures on islands tend to be quite divergent [44], there may have been selection of characters that resulted in prezygotic reproductive isolation and phenotypic diversification of this genus in relation to *Panstrongylus*. Justi et al. [8] suggest that events of vicariancy were the main evolutionary mechanisms that acted in the diversification of the *geniculatus* clade species. The main reproductive isolation mechanisms reported for the Triatominae subfamily were ecological and mechanical isolation [45]. The interspecific mating observed among *Panstrongylus* species (Fig. 2) suggests the absence of mechanical barrier. Based on this, we believe that during the divergence of the crossed species, different selective pressures led to events of genomic reorganization that did not numerically alter the chromosomes (with the exception of *P. megistus* [40]) resulting in total reproductive isolation among the evaluated taxa of this clade.

If it is confirmed that all *geniculatus* clade species are really of a single genus (probably *Panstrongylus*) with convergence in morphological characteristics, this case will provide another example of how misleading morphology-based triatomine taxonomy can be (as recently suggested by Monteiro et al. [10]). This highlights the need to combine different approaches (such as molecular clocks, phylogeography and genomic studies) to understand the evolutionary processes of this important group of vectors.

## Conclusion

Our results demonstrate that different from the other groups of the Triatomini tribe [42], as well as the Rhodniini [42, 46], there are prezygotic barriers that prevent the formation of hybrids in the crosses between the *megistus* and *lignarius* group of the *geniculatus* clade. This confirms the specific status of the crossed species and demonstrates why there are no reports of natural hybrids between them. Based on these results, we suggest that the *geniculatus* clade may represent an important evolutionary model for Triatominae, highlighting the need for new studies with greater sample effort for the *geniculatus* clade (grouping the 17 species of *Panstrongylus* and the three of *Nesotriatoma* [1–3]).

## Data Availability

All relevant data are within the manuscript.
